# Paralysis

**Published:** 1846-04

**Authors:** 


### PART VI.—ECLECTIC DEPARTMENT.

1. *Paralysis.*—The following remarkable case of paralysis, is extracted from the thirteenth annual report of the Massachusetts State Lunatic Hospital, published in November last, by Samuel B. Woodward, M.D., the able and accomplished physician to the institution. It is related to show the derangement of mind, from disease of the external senses, and is interesting, both in a pathological and psychological point of view. Since the publication of the report we learn that the unfortunate sufferer has been relieved by the hand of death. E.

E. A. M., an orphan girl aged 15 years, was admitted to the Hospital, December, 1844. She had had chorea, and has been for sometime melancholy, and effected with headache and great distress in the eyes, which, at such times appeared prominent and distorted. When the paroxysms of headache were over, the eye assumed nearly a natural appearance. For some time she apparently improved, the paroxysms were less severe, and her general health rather better. At this time she began to occupy herself in active domestic employment, she was most of the time cheerful, and we had strong hope of a radical amendment.

These paroxysms did not wholly subside, and when they did occur, she was a great sufferer, and felt gloomy and desponding. She had amenorrhœa, and was inclined to constipation, had bad appetite and sleepless nights. In the intervals of these paroxysms, she was cheerful and active, but would usually say her head ached if she was questioned as to her health.

Some time in March, the disease manifestly increased, the paroxysms became more severe and protracted, and the gloom and despondency increased. In the latter part of April her sufferings were greatly aggravated, the suffering in her head became agonizing, and her sight was greatly impaired. She took no notice of those about her unless touched by them, when she seemed much frightened, but did not speak. During the month of May, she was extremely ill, confined most of the time to her bed. She had severe spasms and excessive head ache, laid nearly senseless, moaning and suffering extremely, often tearing her hair and beating her head with great violence. With the exception of moving her hands, she seemed entirely paralyzed. In this state she continued some weeks apparently near dissolution. She took very little food and emaciated rapidly. Early in June she recovered her speech, and we then ascertained that she was entirely blind and deaf, and had lost the senses of smell and taste. She was entirely unconscious that she was at the hospital, talked to her brothers and sisters as if they were present, and complained in the bitterest terms, that they would not answer her questions, or in any way communicate with her. She had no idea that she was deaf, and it was truly heart-rending to hear her exclaim, "why will you not talk to me—all is silent as the grave—what have I done that you will not speak to me?" For hours together she would address her friends in the most plaintive and imploring language, begging for one word, even if it was that of unkindness. At times she imagined she was a captive among some barbarous people who could not understand her language, and frequently asked if there were no missionaries among them who could serve as interpreters.

While in this condition, after trying various remedies it was thought advisable to try the effect of galvanism. The battery was got ready and the fluid applied gently to her hands, when she became agitated in the most extraordinary degree, her countenance flushed, her eyes glared open and her expression the wildest conceivable, while she continually screamed, "don't bury me alive, don't bury me alive—I am not dead, I am not dead." Never did I witness such a scene—every avenue of communication with her was cut off, and for twelve hours she screamed without intermission, declaring that she was not dead, and begging most imploringly that we would not bury her alive. Too late we found that we had excited a storm which we could not calm; there was no way in which we could soothe or pacify her in the least degree. When completely exhausted, she became quiet and slept. For some days she would start, and appear frightened by the slightest touch. She gradually got better of this excitement and appeared much as she had done for some weeks previous. During all this time, she did not recollect any thing of the hospital or its inmates. She called those about her by the names of her absent friends, myself and assistant she recognised as physicians who had attended her some years previous; her nurse she called by the name of her sister. When I visited her, she would say, "Doctor, why do you not speak to me, you used to be so kind and pleasant, and now you are as silent as the rest; do Doctor, speak one word to me." She improved slowly and favorably till about the middle of June, had taken morphine, submuriate of mercury, the arsenical solution and some laxative medicines, had also had blisters on her temples. Much of the time she swallowed with difficulty, so that food and medicine were given irregularly. On the 17th of June, she again lost her speech, but communicated in a very limited manner by general signs. If thirsty, she would place her fingers on her lips; if she wanted air, she would make the motion of the fan. With this increase of symptoms, she had frequent pulse, dry skin, dry tongue, soreness of the throat, and great difficulty in swallowing. On the 20th of June, for the first time during this long illness, she seemed to have some idea of the hospital, and those who resided here, and by some external mark or peculiarity of dress, recognised a few individuals with whom she had previously been intimately acquainted.

When she first came to the hospital, sad and unhappy, she and another young lady who was equally melancholy, were in the practice of sitting together in their rooms and weeping as they rehearsed their grief. Ascertaining this to be the case, I directed their attendants to prevent such interviews alone, and separate them if thus found talking together. To avoid scrutiny, they learned the manual alphabet and communicated with each other on their fingers quite freely. This was unknown to me at the time, but now it was thought of by the young lady, who was nearly recovered, and she made an attempt to communicate with her upon her fingers. After a time the experiment succeeded, and she learned for the first time that she was deprived of her external senses.

She thought that she had been for a long time in a dark, dismal place, where the people did not talk, and where they drank but did not eat. Early in July, she again lost her speech for forty-eight hours, and during that time was exceedingly sensitive and easily agitated. The slightest touch would alarm her, and bring on trembling; fanning agitated her, and the least motion of the bed clothes would excite and disturb her.

Till the sixth of July, the senses remained lost. Various experiments were tried with her to satisfy us that they were entirely gone. She drank vinegar as water, took a tea spoonful of cayenne pepper as she would take so much bread, without noticing it in the least. On the 7th of July, the sense of smell was suddenly restored and she enjoyed the fragrance of flowers and other perfumes. This was the first restoration of any of the external senses, and it remained but a short time. For some days she had been very comfortable and seemed to be improving favorably. On the night of the 12th, the nurses were awakened by her groans, and found her in a state of great trepidation and alarm, which was only increased by every attempt to soothe her. When any one touched her, she would spring away in great fear, her eyes staring, and her whole frame trembling with agitation. In the afternoon of the next day, she became more composed and was able to recognize a few friends, but had entirely lost her speech. When she recognized any one, she would seize hold of them with a convulsive grasp, and by familiar signs of recognition, and great animation of countenance, would show the joy and satisfaction she experienced in meeting them. When these paroxysms have left her, she has usually supposed that she has been away to some dismal place, "down, down, down." When she meets her friends after the illness has passed, she says with great animation and expressions of delight, "got back, got back." At this time, she communicated readily with her fingers, and many of her friends learned her language. Her sense of touch was very acute. She read with ease the lettering on books, and the books for the blind. Her mind was very active, and her memory retentive. She walked about with some assistance, and gains strength and health between these paroxysms. She talked much with her friend J——, who had recovered, and was employed to converse with and take charge of her. When she is in one of her paroxysms she is dull, understands but little, and often loses her memory of every thing. Her smell repeatedly returned for a short period, but was soon gone again; so far as we have been able to discover, she has been totally blind from the first, and deaf since the first loss of hearing. She has repeatedly come out of these paroxysms with some delusion upon her mind. Once she supposed that her mother had visited her and brought her ornaments, which had been lent her; these she afterwards claimed as her own. For a long time, this impression remained, though her mother died when she was two years old. In the intervals of these paroxysms, she is sensible and rational, very quick in her discernment, and greatly disposed to mirthfulness.

Early in August, when she was in this comfortable state, she was frightened by some one coming to her room and taking hold of her. She was so much agitated by this, that the slightest touch, for a number of days, would alarm her. Her trepidation on this occasion was so great that she could hardly be persuaded to take her food or drinks, every communication frightened her so much. She again recovered from this fear, and was cheerful and happy. She took much pleasure in knitting and was very industrious.

On the 12th of August her friend J. left her. She had been most kind and faithful to her, and her departure was a cause of great grief; they were mutually attached, and both felt sad at the separation. Her attachment to J. was very strong, and whenever her name was communicated to her, her countenance brightened and she was full of animation and joy. Soon after this good friend left her, our patient was removed to the family apartments, where she has since remained.

On the 14th of August she had another paroxysm, which is thus described by one who was with her: "Her face assumed a singular expression, her eyes rolled wildly, and when I attempted to speak with her, I found she did not notice at all. She kept my hand but would suffer no other hand to touch her without an expression of horror. She trembled constantly, started frequently, and seemed to be in the greatest fear. In about an hour she became more calm and slept. It was thought best to place her in bed, as she was not in a comfortable position. When touched for this purpose, she manifested the greatest agitation, and screamed in the most frightful manner, resisting with all her power. When finally placed in bed, she crouched close to the wall, trembled so as shake the bed, and with every breath continued those dreadful screams. After an hour she became more quiet and slept again. When she waked she felt about to ascertain where she was, examined the hands of her watch to tell the time, and recognized the friend who was with her. She moved her hands about in an awkward manner as if she had some idea of having communicated with them. When we attempted to talk with her in the usual manner we ascertained that she had forgotten her alphabet; she had also forgotten how to knit, though she was knitting when the paroxysm came on. After a few attempts she succeeded in writing her wants on the slate, and in the course of the day learned her alphabet again, and was able to knit." As usual, she supposed that she had been away during the paroxysm, said she was "so glad to get back," and would never go away again. She did not enquire for any one, and was much afraid of being touched. She had been intimately acquainted with many persons in the house, but now seemed to have forgotten them all, and for a considerable time could not be made to understand who they were. After a few days she recollected that there were three physicians here, and gradually regained her knowledge of other friends.

For many days in the latter part of August, she was quite ill, suffering from palpitation, head ache, and severe neuralgia. After these symptoms had continued some days, she began to lose the use of her lower limbs; they were very painful, and she attempted to walk, tottered and repeatedly fell. Early in September she lost the use of them entirely, and all sensibility in them. For a time they were perfectly cold and white, as if dead, and pins or needles thrust into them produced no impression. They have since appeared more natural, but are yet insensible. At this time she very suddenly regained her senses of smell and taste, and was made very happy by it, thought her other senses would soon be restored, and indulged in many pleasing anticipations. Soon after this she began to articulate a few words, though in an imperfect manner; she was not conscious of it, and when told that she had really spoken, her delight was unbounded, she repeated the words continually, and daily added to their number.

She was now very happy, could taste, smell and talk considerably. Her feeling was also very acute, so that she could not only read the books for the blind, which she did readily, but coins, seals and engraved visiting cards. She was very industrious and employed herself in knitting purses, making worsted mats and sewing. The articles thus made she sold to visitors, and took great pleasure in counting her money and shewing it to her friends. She prepared some articles to be exhibited at the Fair in October, and was much delighted when she learned that she had obtained a premium for them. She was much interested in all accounts of the Fair, and the descriptions of the articles there exhibited. The evening after the Fair she attended a dancing party, which was got up for the patients, and though she could neither see the dancing nor hear the music, she enjoyed it greatly. She could feel the motion of the floor, and took the hands of the dancers as they passed her. Examined their dresses, inquired of all her associates who they had for partners, and seemed quite the happiest person in the room.

At this time she wrote to her brother that she was "as happy as the day is long." She remained in this state of enjoyment and good health till the 20th October, when she again became ill and for several days was in intense pain. She had severe palpitation, and the pain about the heart was very violent, her eyes were much swollen and inflamed, and the distress in her head very great. Much of her pain seemed to be neuralgic, and she suffered apparently beyond human endurance. Large doses of morphine only afforded relief, and these often repeated. For many days her life was in jeopardy, and it seemed hardly desirable that she should again recover. Her distress gradually subsided, but we found that she had again lost her speech, and with it, all feeling in her hands; they were entirely useless. Her situation was now more deplorable and hopeless than ever. The only medium by which she had been able to communicate with those around her, was now withdrawn, and existence was a burden. She could not speak as in her first attack, for she was now dumb; she could not recognize her friends by the sense of touch, for her hands were paralysed. She could see no one, hear no one, feel no one, and she constantly suffered the most agonizing pain. Her friend watched her with increasing vigilance and solicitude, and as she was relieved from suffering, she commenced teaching her an alphabet on her face. By untiring perseverance, she succeeded in communicating to her simple ideas in this novel manner. While this experiment was going on successfully, the sensibility of her hands was at once partially restored, and she was again able to converse in her usual manner.

From this severe illness she gradually recovered to her former condition, except that the acute sense of feeling which she had long had in her fingers, did not return, and she has since been unable to read even the raised letters of the blind.

During the month of November, she has been very comfortable most of the time, has had considerable head ache, and gets easily fatigued, yet she is ever cheerful and happy. For some days previous to the annual thanksgiving, she was anticipating the pleasures and festivities of that occasion, and when the day arrived she was full of enjoyment. She dined with her friends, and in the evening enjoyed the dance as much as before. She now spends her time in knitting, sewing, writing and playing games. In her chair on castors she moves about from one place to another, enjoying the salutations which she every where meets, and though quite blind, deaf and dumb, and unable to walk or read, she has much real pleasure, is ever patient, cheerful and thankful to those friends who meet her with affectionate kindness and sympathy, and grateful to her Heavenly Father for so many favors and blessings in the midst of all her privations and sufferings.

*Archiv. fur Physiol. und Pathol. Chemie and Microsc., Heft I.

